# Improper monitoring and deviations from physiologic treatment goals in patients with brain injury in the early phases of emergency care

**DOI:** 10.1007/s10877-019-00455-0

**Published:** 2020-01-14

**Authors:** Siri Kojen Andersen, Ragnhild Hustveit, Erlend Frøland, Oddvar Uleberg, Andreas Krüger, Pål Klepstad, Trond Nordseth

**Affiliations:** 1 Department of Circulation and Medical Imaging, Faculty of Medicine and Health Sciences, Norwegian University of Science and Technology, P.O. box 3250, Sluppen, 7006 Trondheim, Norway; 2 Department of Emergency Medicine and Pre-hospital Services, St. Olav`s University Hospital, 7006 Trondheim, Norway; 3 Department of Research and Development, Norwegian Air Ambulance Foundation, P.O.Box 6770, 0130 Oslo, Norway; 4 Department of Anaesthesia and Intensive Care Medicine, St. Olav University Hospital, 7006 Trondheim, Norway; 5 Regional Centre for Health Care Research, St. Olav University Hospital, 7006 Trondheim, Norway; 6 Department of Anesthesia Molde Hospital, Møre og Romsdal Hospital Trust, 6412 Molde, Norway

**Keywords:** Pre-hospital care, Physiological deviations, Traumatic brain injury, Cardiac arrest, Intracerebral bleeding, Physician-staffed Emergency Medical Service

## Abstract

Severe traumatic brain injury (TBI), out-of-hospital cardiac arrest (OHCA) and intracerebral- and subarachnoid hemorrhage (ICH/SAH) are conditions associated with high mortality and morbidity. The aim of this study was to investigate the feasibility of obtaining continuous physiologic data and to identify possible harmful physiological deviations in these patients, in the early phases of emergency care. Patients with ICH/SAH, OHCA and severe TBI treated by the Physician-staffed Emergency Medical Service (P-EMS) between September and December 2016 were included. Physiological data were obtained from site of injury/illness, during transport, in the emergency department (ED) and until 3 h after admittance to the intensive care unit. Physiological deviations were based on predefined target values within each 5-min interval. 13 patients were included in the study, of which 38% survived. All patients experienced one or more episodes of hypoxia, 38% experienced episodes of hypercapnia and 46% experienced episodes of hypotension. The mean proportion of time without any monitoring in the pre-hospital phase was 29%, 47% and 56% for SpO_2_, end-tidal CO_2_ and systolic blood pressure, respectively. For the ED these proportions were 57%, 71% and 56%, respectively. Continuous physiological data was not possible to obtain in this study of critically ill and injured patients with brain injury. The patients had frequent deviations in blood pressure, SpO_2_ and end tidal CO_2_-levels, and measurements were frequently missing. There is a potential for improved monitoring as a tool for quality improvement in pre-hospital critical care.

**Electronic supplementary material:**

The online version of this article (10.1007/s10877-019-00455-0) contains supplementary material, which is available to authorized users.

## Introduction

Severe traumatic brain injury (TBI), out-of-hospital cardiac arrest (OHCA), intracerebral- and subarachnoid haemorrhage (ICH/SAH) are critical conditions associated with a high risk of poor outcome. The estimated mortality rates for these conditions are 45%, 90% and 50%, respectively [1–4]. Severe TBI, OHCA and ICH/SAH may lead to circulatory disturbances causing reduced perfusion of the brain, with increased risk of secondary brain damage [5, 6]. In the pre-hospital environment, these patients may be especially vulnerable when exposed to unfavourable physiological factors such as airway problems, hypoxia and hypotension—all factors that may negatively affect survival and cerebral outcome [7]. Physiological variables such as blood pressure, pulse oximetry (SpO_2_) and end tidal CO_2_-levels need to be well regulated to prevent hypoxia, reduced perfusion and increases in intracerebral pressure (ICP). The patients should ideally be monitored continuously throughout the pre- and early in-hospital course of treatment [8]. A previous study on physiologic deviations in patients with TBI in the ICU showed that 35% of the patients experienced hypotension during the first 2 days after admittance, while 20% experienced episodes of hypoxia [9]. Few studies have described the extent of physiological deviations throughout the pre-hospital and early in-hospital phase, which is unfortunate as these types of deviations may negatively affect patient outcomes [10]. Both physiologic monitoring and adherence to treatment goals are challenging tasks in the pre-hospital and early in-hospital phase [11]. Thus, unfavourable physiologic parameters may be inadequately observed and documented [12]. In this study, we aimed to investigate the feasibility of obtaining continuous monitoring and identification of physiological deviations in patients with acute brain injury, in the early phases of emergency care.

## Methods

### Study setting

The study was conducted as a prospective observational study at the Physician-staffed Emergency Medical Services (P-EMS) in Trondheim, Norway. This service is part of the national air ambulance services offering specialized medical assistance to critically ill or injured patients [13]. P-EMS Trondheim mainly covers the counties of Trøndelag, Møre and Romsdal in Central Norway, with a population of approximately 750,000 [14, 15]. The study was assessed by the Regional Ethics Committee (reference number 2016/845/REK Midt), which waived the need for patient consent according to the Norwegian Health Research Act.

The treatment and monitoring on scene is initiated and guided by standard operating procedures at the physician’s discretion. Patients are mainly transported by ambulance or helicopter to the Emergency Department (ED) of St. Olav’s University Hospital, which is a 700-bed tertiary care hospital located in Trondheim [16]. After initial care and assessment, the patients included in this study were admitted to either the mixed-case Intensive Care Unit (ICU), the neurosurgical ICU or the cardiac ICU.

### Data collection

Adult and pediatric patients treated by the P-EMS with a suspected acute brain injury and a reduced level of consciousness were considered for inclusion. This included OHCA with return of spontaneous circulation (ROSC) or a suspected acute neurological condition, i.e. ICH, SAH or severe TBI. Patients treated by the P-EMS on scene and admitted to St Olav’s University Hospital between September 1st and December 5th, 2016 were considered for inclusion. Inclusion was based on clinical symptoms and/or findings suggesting acute brain injury, thus patients with convulsions were included.

Patients transferred between hospitals and those who died prior to hospital arrival were excluded. Missions not started or aborted en-route due to operational issues (e.g. weather) or lack of need for specialized physician services were excluded. Patients who were not attached to a monitor during the prehospital phase of treatment were excluded. Diagnosis of the initiating event was established during hospital follow-up.

Demographic and in-hospital clinical data were collected from the patients’ electronic medical records (DocuLive, Siemens Nixdorf Information Systems, Oslo, Norway), including further in-hospital course of events and relevant comorbidities. Patients considered for inclusion were physiologically monitored from the scene of injury/illness until the first 3 h of treatment in the ICU, using the Tempus Pro Monitor (Remote Diagnostic Technologies, Basingstoke, UK) and/or Corpuls3 (GS Elektromed. Geraete, Kaufering, Germany). The monitors collected data on physiologic variables including blood pressure, end tidal CO_2_-levels and SpO_2_. Analysis of signal quality was not performed. The Tempus Pro Monitor was applied for study purposes and only used by the P-EMS. The monitor was connected until arrival in the ICU when applied. The Corpuls3 monitor was used by both ground ambulances and the P-EMS, and was sometimes detached when care of the patient was transferred to the P-EMS. After arrival to the ED, the patients with a Corpuls3 monitor were connected to in-hospital monitor. This because the Corpuls3 was the standard monitor in clinical use, thus it could not be left in the hospital. Data from the in-hospital phase of the treatment were collected by manual registrations in the ED and during in-hospital transport, and from the ICU electronic records (Critical Care Manager, Picis, Wakefield, MA, USA).

Episodes of hypercapnia, hypoxia or hypotension were defined according to the treatment goals given in Table 1. Time untreated was defined as the time before arrival of EMS or P-EMS. The number of deviations was defined as the number of 5-min-periods where any deviations from the treatment goals were registered. The patients were classified as unmonitored if no physiologic measurements were performed in a given 5 min-period. Patient comorbidity was assessed using the Charlson Comorbidity Index [17]. Patients received follow-up to hospital discharge or death.

**Table 1:** Physiologic target values for the patients included

Physiologic parameter	Target value
Systolic blood pressure	≥ 90 mmHg
SpO_2_	≥ 93%
End tidal CO_2_	< 45 mmHg/6 kPa

### Statistics

Data was analyzed using the software Excel (Microsoft Corporation, Redmond, WA, USA) and the software Matlab (The Mathworks, Natick, MA, USA). The degree of monitoring and whether or not the monitored variable deviated from the predefined treatment goals were visualized applying the package ‘TraMineR’ with the software R version 3.6.0 [18, 19].

## Results

**Fig. 1:**
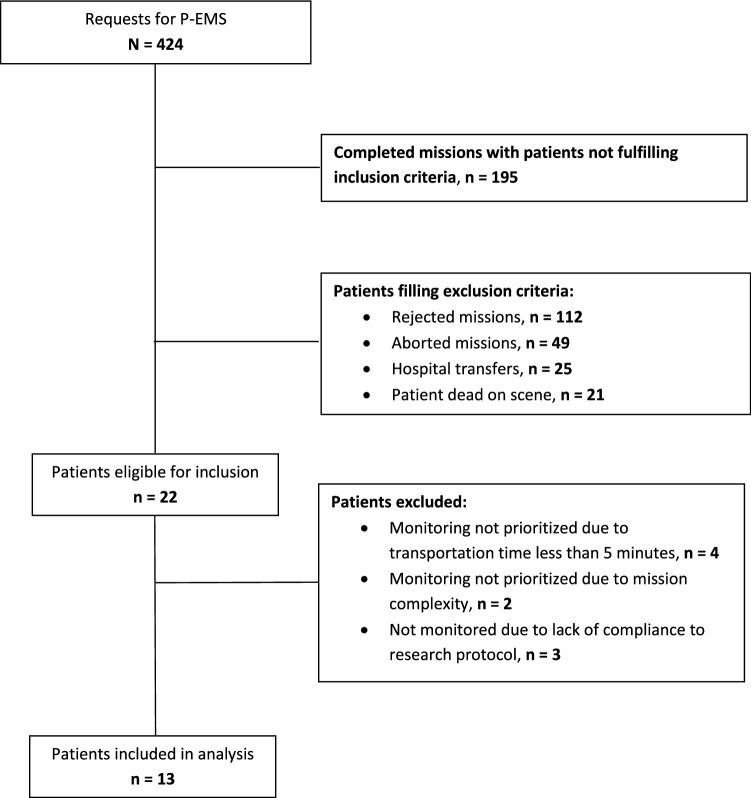
Flowchart of patient inclusion during study period (September–December 2016)

There were 424 requests for P-EMS during the 3 months period of inclusion. A flowchart of patient inclusion and exclusion is demonstrated in Fig. 1. Thirteen patients were included in the study, of whom five survived to hospital discharge (38%). Baseline characteristics for the patients included are presented in Table 2. The median age was 67 years (range 1–89 years). The most frequent condition was OHCA (six patients). The patients had an overall low comorbidity, with a maximum Charlson Comorbidity Index score of three.

**Table 2:** Baseline demographic data for the patients included

	Condition	First GCS	Time untreated (min)	Time unmonitored (min)	Transportation time (min)	Charlson Comorbidity Index
1	Traumatic head injury	3	8	14	25	0
2	OHCA	3	12	3	29	3
3	Strangulation with ROSC	3	8	3	17	0
4	SAH	7	9	3	33	1
5	Cerebral haemorrhage	3	12	19	6	0
6	Prolonged convulsions	–	12	6	17	0
7	OHCA	–	7	3	4	0
8	OHCA	3	8	0	33	0
9	Drowning and CPR^a^	–	78	18	–	0
10	OHCA	3	7	29	9	0
11	Cerebral haemorrhage	4	14	3	15	1
12	OHCA	3	6	–^b^	35	3
13	Traumatic head injury	3	12	–^b^	3	0

*OHCA* out-of-hospital cardiac arrest, *ROSC* return of spontaneous circulation, *CPR* cardiopulmonary resuscitation

^a^CPR given during transport. Data on transportation time is missing

^b^Data from the monitoring done by the ground-EMS personnel could not be extracted

**Fig. 2:**
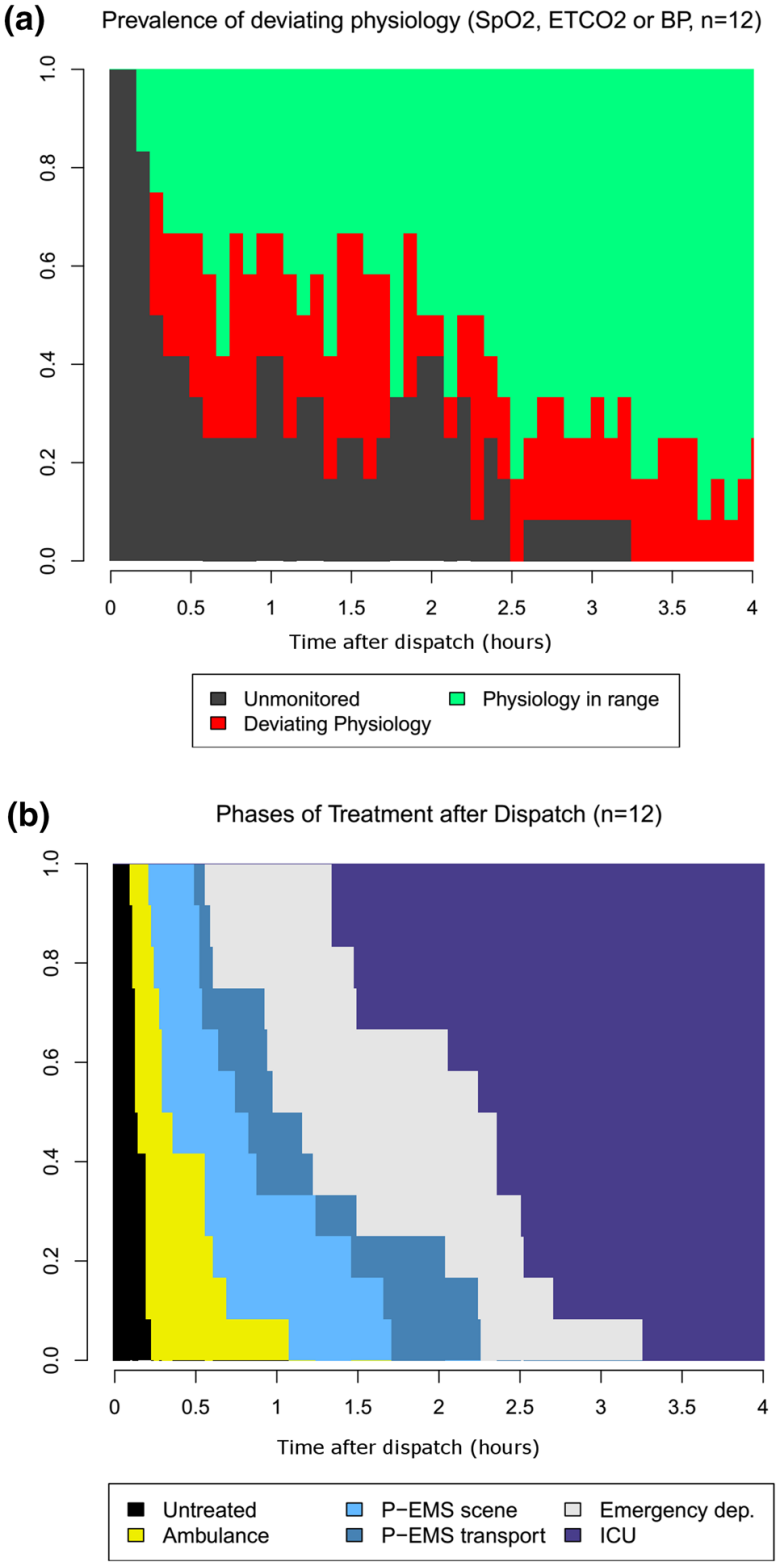
The upper figure **a** demonstrated the proportion/prevalence of patients being either unmonitored (grey), being monitored and have deviating physiological values (red) or being monitored and physiologic values in target range (green), for the first 4 h of treatment. The physiologic variables were considered to deviating (red) if at least one of the parameters SpO_2_, endtidal CO_2_ (EtCO_2_) or systolic blood pressure (BP) were out of range in a given 5-min interval. The lower figure **b** demonstrates the proportion of patients with time being either untreated (black), treated by ground ambulance (yellow), treated by P-EMS (blue), being treated in the emergency department (light grey) or in the ICU (lilac). One patient was excluded from the analysis due to severe hypothermia, giving imprecise measurements

The physiological observations from the pre-hospital, ED and ICU were successfully combined to one time axis. For operational reasons, one patient had a prolonged time to start of prehospital treatment (78 min) and was not included in the analysis of deviations from treatment goals due to severe hypothermia. The observed deviations from the defined treatment goals and the extent of monitoring for at least one of the physiologic variables are visualized in Fig. 2a for the first 4 h of treatment. The distribution of patients being in different phases of treatment with time is demonstrated in Fig. 2b. Physiologic deviations and the extent of monitoring for the individual physiologic parameters are demonstrated in Supplementary Fig. 1.

SpO_2_ was the physiological variable with the highest proportion of deviations during the pre-hospital phase, accounting for 56% of the total number of deviations. In the ED, systolic blood pressure was the variable with the highest proportion of deviations, (47%). All of the patients experienced one or more episodes of oxygen desaturation, 46% experienced episodes of hypotension, and 38% experienced episodes of hypercapnia. In two patients with ICH and TBI, there was observed an EtCO_2_ of less than 4.5 kPa (hypocapnia) for 38 min and 14 min, respectively. The remaining five patients with ICH/SAH or severe TBI had no measurements of EtCO_2_.

The mean proportion of time without any monitoring in the pre-hospital phase was 29%, 47% and 56% for SpO_2_, end-tidal CO_2_ and systolic blood pressure, respectively. In the ED the corresponding proportions were 57%, 71% and 56%, respectively.

The mean time untreated by health care personnel was 15 min (range 6–78 min). Ground-EMS personnel arrived at the scene prior to P-EMS in 12 patients. In two patients, ground-EMS personnel did not initiate any monitoring. The mean time unmonitored was 9 min (range 0–29 min). The mean duration of transport from scene to hospital was 22 min.

## Discussion

Our study demonstrates that it is feasible to obtain physiological data from several data sources during acute care trajectories until definitive care in the ICU. In this study, physiological variables from the pre- and in-hospital phase for patients with ICH/SAH, OHCA and severe TBI were merged, offering a possible continuously sampling of data in the initial phase of critical illness or injury. However, the high number of 5-min intervals without measurements of important physiologic variables does not justify the use of the term continuous physiological data in this study. Frequent deviations from physiological treatment goals were observed, but the study was too small to assess any possible effects on patient outcome.

It may be challenging to establish and maintain monitoring, especially when patients are handled between different health care providers. Inadequate documentation in this phase has been demonstrated to be associated with increased mortality, although this may be confounded by the severity of illness or injury [20]. The pre-hospital operational setting with bad weather, temperature changes, and movement may contribute to the lack of monitoring. In addition, prioritization of immediate treatment over monitoring and the use of several different monitors along the treatment trajectory may affect the degree of monitoring. Clinical and operational decisions are continuously made, balancing the need for rapid transfer to hospital versus establishing advanced therapy before and during transport. For the patient groups included in this study, an optimization of oxygenation and circulation on site and during transport may have a better impact on patient outcome than rapid transfer to hospital. Although most patients had arrived at hospital within 2 h after dispatch, lack of monitoring and deviations from treatment goals continued to be an issue.

For some patients, monitoring was not initiated by either P-EMS or ground-EMS before a considerable amount of time had passed and was often incomplete. Moreover, it occurred that patients were detached from the monitor leaving possible physiological deviations undetected at several occasions. This may be related to that Ground-EMS and P-EMS used separate monitors. However, it is unlikely that detachment and re-attachment of equipment should take more than a couple of minutes in a normal operational setting, suggesting that other interventions may have been prioritized [21]. Occasionally, physiological variables in the ED were not registered. To obtain accurate documentation in the ED and during radiologic examinations may be challenging but favorable [22, 23]. Our observations agrees with a study by Chen et al. on in-hospital documentation of vital signs, which illustrated the difficulty of maintaining continuous monitoring of critically ill hospitalized patients [24]. As we have demonstrated in Fig. 2b, the proportion of patients residing in the ED (i.e. the relative height of the grey field) was highest between 1 and 2 h after dispatch (20–50% of patients). During this period, the overall proportion of deviations reached nearly 40% (i.e. the relative height of the red field in Fig. 2a), and this was mainly due to hypoxia and hypotension (Supplementary Fig. 1). The low number of patients precludes an in-depth analysis of these issues.

The degree of physiological deviations experienced by the patients may be due to insufficient treatment provided or difficulties providing this given their critical condition. SpO_2_ was the variable that deviated most frequently in the pre-hospital phase. This is unfavorable, as hypoxia in the acute phase is known to affect the outcome negatively [7]. SpO_2_ was also the most frequently monitored variable in the pre-hospital phase in our study. For some of the patients, monitoring of SpO_2_ was discontinued when patients were hypoxic, possibly leaving the patients hypoxic for an unknown amount of time. However, some missing SpO_2_ measurements might be related to the technology itself, as reduced blood perfusion might impede SpO_2_ measurements.

Systolic blood pressure had the highest proportion of deviations in the ED, and hypotension is associated with increased mortality after brain injury [25]. Insufficient monitoring of systolic blood pressure is also documented in other studies [11, 21]. However, in the majority of patients, only non-invasive blood pressure monitoring was performed. The accuracy of this method is uncertain in hypotensive patients, and clinical signs may be considered as more useful by the clinicians—a possible cause for lack of measurements [26]. After admittance to the ICU, wide fluctuations in systolic blood pressure were observed, which might occur due to patients being in an unstable circulatory status before and during initial resuscitation.

Exposure to hypercapnia after brain injury is associated with a poor clinical outcome [27]. In four patients, potential deviations in end tidal CO_2_ were not registered due to lack of monitoring. EtCO_2_ had the lowest proportion of deviations in the ED, but also the highest proportion of missing data. However, in one patient (patient 2) where EtCO_2_ was monitored continuously throughout the pre-hospital phase, 11 deviations in EtCO_2_ were observed prior to hospital arrival. This agrees with the general self-fulfilling observation that the variables that deviated most frequently were the variables most often registered.

This study has several limitations. First, few patients were included in the study and they suffered from a wide range of medical conditions. This makes it difficult to draw specific conclusions regarding exposure to adverse physiological events and long-term outcome. Second, this was a one-center pilot study giving preliminary information needed before commencing further studies. Third, non-invasive systolic blood pressure was documented in the pre-hospital phase and invasive systolic blood pressure in the early in-hospital phase, which may cause variations in the results. Finally, with respect to EtCO_2_ measurements, we defined hypercapnia (> 6 kPa) as a deviation from optimal physiology. Hypocapnia (< 4.5 kPa) may also be harmful in these groups of patients. We considered possible gas leakage when using supraglottic devices, differences in end-tidal and arterial CO_2_ and poor circulation in patients with return of spontaneous circulation (ROSC) as potential sources of bias in this regard. For these reasons, we did not report the extent of hypocapnia in ROSC patients, and only two out of seven non-ROSC patients in this sample had measurements of EtCO_2_ limiting further exploration of this issue.

## Conclusions

Continuous physiological data was not possible to obtain in this study of critically ill and injured patients with brain injury. When physiologic measurements were made, we observed frequent deviations in blood pressure, SpO_2_ and end tidal CO_2_-levels from first contact in the field to arrival in the ICU. Physiological monitoring was missing for sustained periods of time, both in the pre- and in-hospital phase. However, to collect precise physiological data profiles may be a promising tool for quality improvement in pre-hospital critical care.

## Electronic supplementary material

Below is the link to the electronic supplementary material.

Supplementary Fig. 1 The upper, middle and lower plots demonstrate the proportion of patients being either unmonitored (grey), being monitored and have deviating physiological values (red) or being monitored and physiologic values in target range (green), for the first 4 h of treatment for the parameters SpO_2_, systolic blood pressure and end-tidal CO_2_, respectively. One patient was excluded from the analysis due to severe hypothermia, giving imprecise measurements. (PDF 141 kb)

## Abbreviations

TBI: Traumatic brain injury
ICH: Intracerebral haemorrhage
SAH: Subarachnoid haemorrhage
OHCA: Out-of-hospital cardiac arrest
P-EMS: Physician-staffed Emergency Medical Service
ED: Emergency department
ICU: Intensive care unit
ICP: Intracerebral pressure
GCS: Glasgow Coma Scale
EMS: Emergency Medical Services
ROSC: Return of spontaneous circulation
REC: Regional Ethics Committee (REC)
