# Supplementary material for: Improper monitoring and deviations from physiologic treatment goals in patients with brain injury in the early phases of emergency care
Source: J Clin Monit Comput. 2020 Jan 14;35(1):147–53. doi: 10.1007/s10877-019-00455-0 (PMC7889683; doi:10.1007/s10877-019-00455-0)
Supplement: Supplementary file 1 — The upper, middle and lower plots demonstrate the proportion of patients being either unmonitored (grey), being monitored and have deviating physiological values (red) or being monitored and physiologic values in target range (green), for the first 4 h of treatment for the parameters SpO2, systolic blood pressure and end-tidal CO2, respectively. One patient was excluded from the analysis due to severe hypothermia, giving imprecise measurements. (PDF 141 kb) [file 10877_2019_455_MOESM1_ESM.pdf]

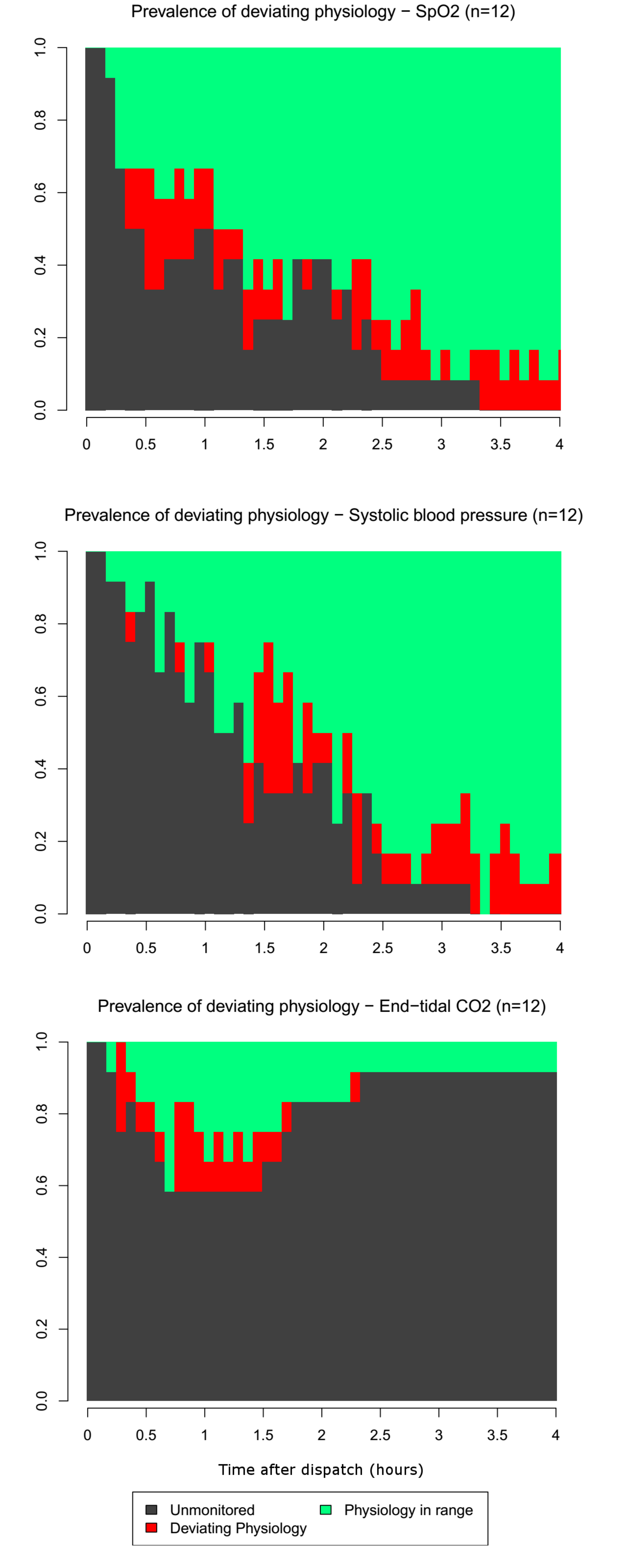

Prevalence of deviating physiology − SpO2 (n=12)
Prevalence of deviating physiology − Systolic blood pressure (n=12)
Prevalence of deviating physiology − End−tidal CO2 (n=12)
0.0
0.2
0.4
0.6
0.8
1.0
0
0.5
1
1.5
2
2.5
3
3.5
4
Time after dispatch (hours)
Unmonitored
Deviating Physiology
Physiology in range
